# Assessment of the Pro12Ala Polymorphism in the *PPAR-γ2* Gene among Type 2 Diabetes Patients in a Nigerian Population

**DOI:** 10.3390/jcm7040069

**Published:** 2018-04-05

**Authors:** Godwill Azeh Engwa, Friday Nweke Nwalo, Venatus Osita Chiezey, Marian N. Unachukwu, Opeolu Oyejide Ojo, Benjamin Ewa Ubi

**Affiliations:** 1 Department of Chemical Sciences, Godfrey Okoye University, Thinkers Corner, Enugu P.M.B 01014, Nigeria; 2 Department of Biotechnology, Faculty of Science, Ebonyi State University, Abakaliki P.M.B 53, Nigeria; ubi.benjamin1@yahoo.com; 3 Department of Biotechnology, Federal University, Ndufu-Alike Ikwo (FUNAI), Abakaliki P.M.B. 1010, Nigeria; friday.nwalo@funai.edu.ng; 4 Department of Microbiology, Godfrey Okoye University, Thinkers Corner, Enugu P.M.B 01014, Nigeria; chiezeyo@yahoo.com (V.O.C.); drunachukwu@yahoo.com (M.N.U.); 5 Diabetes Research Group, Department of Biology, Chemistry and Forensic Science, School of Science, Faculty of Science, University of Wolverhampton, Wulfruna Street, WV1 1LY Wolverhampton, UK; o.o.ojo@uel.ac.uk

**Keywords:** type 2 diabetes, *PPAR-γ2* gene, Pro12Ala polymorphism, obesity, lipid profile indices, Nigeria, genetic association

## Abstract

The association between the Pro12Ala polymorphism of the *PPARγ2* gene, type 2 diabetes (T2D), and obesity in certain ethnic populations has been reported. However, this relationship has not yet been described among diabetes patients in Nigeria. This study investigated the relationship between the Pro12Ala polymorphism in the *PPARγ2* gene, obesity, and lipid abnormalities characterizing T2D among patients in Nigeria. This case-control study recruited 73 T2D and 75 non-diabetic (ND) patients. Demographic and clinical data were collected and blood glucose levels together with serum lipid profile for patients were measured. Pro12Ala polymorphism in the *PPARγ2* gene was genotyped by restriction fragment length-Polymerase Chain Reaction (RFLP-PCR). The *PPAR-γ2* gene (amplicon size = 270 base pair) was successfully amplified for all samples. Following restriction enzyme digestion and analysis by agarose gel electrophoresis, amplicons from samples showed a band of size 270 bp and were of the wild homozygous Pro/Pro genotype. Ala12 variant was totally absent from the study population. Obesity, estimated using Body Mass Index (BMI) and waist circumference (WC), was significantly higher (*p* < 0.05) in T2D patients compared to the non-diabetic patients. More so, the prevalence of lipid abnormalities; hypercholesterolaemia (TC > 200 mg/dL), hypertriglyceridaemia (TG > 150 mg/dL), high HDL (>100 mg/dL), and low HDL (<50 mg/dL) was significantly greater (*p* < 0.001) in T2D patients compared to non-diabetic patients. Results obtained further indicated lack of significant association between *PPAR-γ2* gene polymorphism, T2D, and obesity. However, obesity and dyslipidaemia were strongly associated in T2D patients.

## 1. Introduction

Diabetes is a major threat to humans affecting over 400 million people across the globe [1]. Among the diabetic cases, the majority are due to type 2 diabetes (T2D) [1]. Several factors predispose people to T2D, among which obesity constitutes a major risk factor and is highly associated with the disease [2]. According to a WHO global report, over 650 million people were obese in 2016 [3]. On the other hand, obesity is usually associated with dyslipidaemia, a major lipid abnormality characterised by increased levels of total cholesterol (TC), triglyceride (TG), low density lipoprotein (LDL), and reduced level of high density lipoprotein (HDL). Dyslipidaemia is highly prevalent and associated with T2D. Hypertriglyceridaemia, a component of dyslipidaemia, is known to be the principal lipid abnormality that promotes insulin resistance [4]. Hypertriglyceridaemia is responsible for increased production and decreased clearance of lipoproteins leading to increased levels of free fatty acids (FFA) in the blood stream [5,6].

In a healthy and non-diabetic state, insulin promotes triglyceride storage in adipocytes and prevents lipolysis and release of FFAs. Peroxisome proliferator activated receptor protein (PPAR) is one of the molecules which regulate triglyceride storage in adipocytes. *PPAR* is a nuclear receptor ligand-activated transcription factor commonly expressed in adipose tissue, liver, and heart [7]. The gene exists in three isoforms namely; *PPAR-α*, *PPARβ/δ*, and *PPAR-γ*. *PPAR-γ* has been shown to be the isoform that is functionally related to diabetes [8] as it plays a major role in glucose metabolism, differentiation of adipocytes, regulation of lipid metabolism, promotion of insulin sensitivity, and has beneficial effects on plasma lipids [9,10]. Synthetic ligands of *PPAR-γ* such as rosiglitazone and pioglitazone have been reported to facilitate increased serum HDL and reduced plasma triglyceride levels [11]. 

Several polymorphisms have been identified in the *PPAR-γ2* gene [12]. The most important is the Pro12Ala polymorphism on exon B of the *PPAR-γ2* gene. This polymorphism is defined by a CCA to GCA mutation on codon 12 changing a proline (Pro) to an alanine (Ala) [13]. Previous studies have shown the *Ala* allele to confer reduced risk of diabetes development among ethnic groups, such as Japanese-Americans [14,15]. The frequency of *Ala* allele among Asians and Africans is low (1–3%) with most individuals having the *Pro12* allele [16]. Though an expanding body of evidence reports the relationship between the Pro12Ala polymorphism of the *PPARγ2* gene and T2D and/or obesity in some African populations [17,18,19], none of these studies assessed the polymorphism within a Nigerian population. 

## 2. Materials and Methods

### 2.1. Study Population and Design

This study was part of an ongoing case-control study involving T2D and non-diabetic (ND) patients at Enugu State University Teaching Hospital (ESUTH) in Enugu, Nigeria. Ethical approval was obtained from the ethical committee at ESUTH and the study was conducted in accordance with the Helsinki Declaration. Written informed consent was obtained from all patients before recruitment into the study. Patients above 30 years of age, who have not eaten for the past 12 h (overnight fasting), without any critical or emergency health conditions or complications and not admitted at the hospital were recruited for the study. Breastfeeding and/or pregnant women as well as HIV positive patients were excluded from the study. T2D patients were those with at least a one-year history of the disease, diagnosed according to the International Diabetic Federation (IDF) criteria [20]. ND patients, who served as the control, were those without hyperglycaemia or diabetes. 

### 2.2. Data Collection and Laboratory Analysis

Demographic information of patients including age and sex was obtained while the systolic blood pressure (SBP) and diastolic blood pressure (DBP) were measured using an automatic sphygmomanometer. Height, weight, and waist circumference (WC) of the patients were measured and the Body Mass index (BMI) calculated. WC and BMI were used to classify obesity according to WHO standard. After interview, 2 mL of blood was collected from each patient, transferred into EDTA free tubes and the fasting blood sugar (FBS) was measured using an Accucheck glucometer (Roche Diabetes Care, Inc., Burgess Hill, UK) according to the glucose oxidase enzymatic method by Trinder [21]. Blood samples were then centrifuge at 5000 rpm for 10 min and serum was obtained for the determination of TC, TG, LDL, and HDL using diagnostic kits from Randox Laboratories Ltd. (Co Antrim, UK) following the manufacturer’s recommended procedure. TC was determined according to the enzymatic method of Allain et al. [22], TG was assayed as described by Esders and Michira [23] and HDL was quantified by the precipitation method of Grove [24]. LDL was calculated using the Freidwald’s [25] formula: LDL = TC − (TG/5) − HDL.

### 2.3. Genotyping of PPARγ2 Pro12Ala Variant

DNA was extracted using GeneJET Genomic DNA Purification kit (K0721, Thermo Fisher Scientific Inc., Waltham, MA, USA). Extracted DNA samples were amplified by PCR and analyzed for Pro12Ala polymorphism of the *PPARγ2* gene by restriction fragment length polymorphism-PCR (RFLP-PCR). The PCR amplification was carried out using the forward (5′-GCCAATTCAAGCCCAGTC-3′) and the reverse (5′-GATATGTTTGCAGACAGTGTATC-3′) primers. The PCR reaction mixture contained 12 µL of OneTaq Quick-Load 2X Master Mix with standard buffer (New England Biolabs, Ipswich, MA, USA), 4 µL of each primer (40 uM), and 8 µL of DNA sample (<10 ng) making a total volume of 28 µL. The reaction was carried out under the following conditions: Initial denaturation at 95 °C for 15 min, followed by 34 cycles of denaturation at 95 °C for 1 min, annealing at 56 °C for 1 min and elongation at 72 °C for 1 min, with a final extension at 72 °C for 10 min. The PCR product was visualised in a UV transilluminator (Bio Olympics, Ltd., Thousand Oaks, CA, USA) after electrophoresis on a 2% agarose gel to confirm the presence of the *PPARγ2* gene. Positive PCR products were digested overnight with *Bst* UI restriction enzyme at 37 °C. The reaction mixture was set to a final volume of 20 µL containing 10 µL of amplicon, 0.4 μL of the *Bst* UI (NEB), 2 µL of 10× smartcut buffer (NEB) and 8 µL of sterile water. The digested products were separated by electrophoresis on a 3% agarose gel.

### 2.4. Statistical Analysis

Data were analyzed using Statistical Package for Social Science (SPSS version 16; IBM Analytics, Armonk, NY, USA.). Results were expressed as mean ± Standard Error of Mean (S.E.M). Parametric independent sample *t*-test was used to compare mean differences of the demographic and clinical characteristics such as obesity (BMI and WC) and lipid profile indices (TC, TG, HDL, and LDL) between T2D patients and ND patients. A *p*-value less than 0.05 was considered statistically significant.

## 3. Results

### 3.1. Baseline Characteristic of Participants

One hundred and forty-eight (148) subjects participated in the study. Among these subjects, 73 (49.3%) were T2D patients while 75 (50.7%) were ND patients (control). The demographic and clinical characteristics of the patients are summarized in Table 1. FBS as well as the weight were significantly higher (*p* < 0.05) in T2D patients compared with their non-diabetic counterparts while the height, DBP and SBP did not show any significant differences between T2D patients and non-diabetic controls.

### 3.2. Obesity and Lipid Profile Indices of Patients

BMI and WC were significantly higher (*p* < 0.05) in T2D patients compared to the control (Table 2). Similarly, TC, TG, and LDL levels were significantly higher (*p* < 0.05) in T2D patients. HDL level was significantly lower (*p* < 0.05) in T2D patients compared to non-diabetic participants. The prevalence of obesity (based on BMI and WC) was significantly higher (*p* < 0.05) in T2D patients compared to non-diabetic control participants. Moreover, the prevalence of lipid abnormalities (dyslipidaemia) characterized by hypercholesterolaemia (TC > 200 mg/dL), hypertriglyceridaemia (TG > 150 mg/dL), high HDL (>100 mg/dL), and low HDL (<50 mg/dL) was significantly greater (*p* < 0.001) in T2D patients compared to non-diabetic patients.

### 3.3. Genotyping for PPARγ2 Pro12Ala Polymorphism

**Figure 1:**
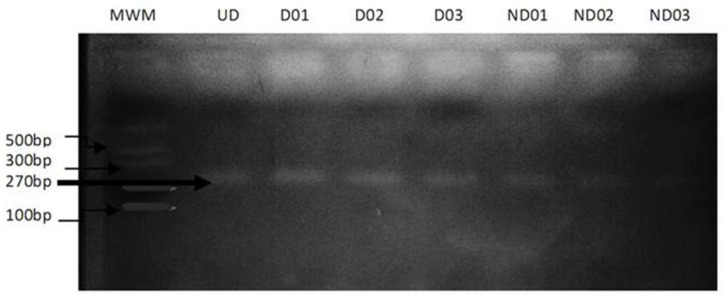
RFLP-PCR of *PPARγ2* Pro12Ala Polymorphism. Samples were analyzed on a 3% agarose gel after restriction digest. D01–D03 = Samples for diabetic patients; ND01–ND03 = Samples for non-diabetic patients; UD = Undigested sample (control); MWM = Molecular weight marker.

The expected DNA fragment of the *PPARγ2* gene after PCR amplification was 270 bp. After a restriction digest, an electrophoretic band of 270 bp was observed for all samples indicating the presence of the homozygous wild-type Pro/Pro genotype which lacks a restriction site (Figure 1) while the mutant homozygous Ala/Ala genotype which has a restriction site was absent as no sample showed band sizes of 227 and 43 bp. Also, the heterozygote Pro/Ala genotype was absent in the study population. As such, the Ala12 variant was totally absent from the study population.

## 4. Discussion

T2D which constitutes about 90% to 95% of all diabetic cases is increasingly on the rise in Africa [26]. This disease has become a major public health problem of concern in Nigeria as recent data has shown Nigeria to have the highest disease burden in the continent of Africa. Incidence of obesity, one of the major risk factors of T2D, and diabetic dyslipidaemia are also on the increase in Nigeria [3]. The important role of *PPARγ2* in the regulation of lipid metabolism coupled with the reported association between *PPARγ* polymorphisms and T2D [15,27,28] further necessitate the investigation of changes in the *PPARγ2* gene among diabetic patients in Nigeria. The Ala variant of the *PPARγ* gene is known to improve insulin sensitivity by altering transcriptional activities and expression of *PPAR-γ* in adipocytes, thereby suppressing lipolysis and release of FFAs while enhancing insulin’s action [29,30]. Though the Pro12Ala variant has shown to be associated with T2D and obesity in various populations across the globe [17], such findings are yet to be revealed in Nigeria. Thus, the Pro12Ala polymorphism and its association with T2D was investigated in this study. Our finding revealed the presence of the homozygous Pro/Pro genotype in all participants; both diabetic and non-diabetic patients, and complete absence of the homozygous Ala/Ala and heterozygous Pro/Ala genotypes. As such, the *Pro12* allele was 100% present while the *Ala12* allele was totally absent in the population. This finding concurs with previous findings in Cameroon which also showed a complete absence of the *Ala12* allele [20] and in Ghana with only one participant having the *Ala12* allele in more than a thousand participants (<0.001%) [31]. This confirms the findings of previous studies which have shown Pro12Ala polymorphism to have a very low frequency (<3%) in populations of African ancestry (African-Americans) [15,16] as well as in typical black Africans population in South Africa and Ethiopian Africans [17]. Cameroon, Ghana, and Nigeria, which present similar genetic profiles, all belong to the Sub-Saharan region of Africa suggesting that areas with similar ethnicity selects particular genetic trait. As such, the prevalence of the *Ala12* allele may vary widely across various ethnic populations. The Caucasian population has been shown to have the highest frequency of this polymorphism with almost 25%, followed by Native Americans with about 10%. Other ethnic populations such as the Japanese, South American, Indian, and Chinese present a frequency less than 4% [14,15]. Findings from this present study may therefore suggest that the *Pro* allele may be the wild type which originated from the African descendants particularly from Sub-Saharan Africa and the *Ala* allele as the mutant type which may have emerged through evolution and environmental changes as the native Africans migrated to other parts of the world. 

A recent meta-analysis study showed the *PPAR-γ2* Pro12Ala polymorphism to be associated with obesity risk, suggesting that the *Ala* allele could increase obesity susceptibility particularly in Caucasian and Asian populations [32]. Thus, it was also of interest to investigate the influence of the Pro12Ala polymorphism on obesity and lipid abnormalities in T2D subjects. Though obesity indices (BMI and WC) were significantly higher (*p* < 0.05) in T2D patients compared to the control and also, dyslipidaemia characterised by elevated TC, TG, and LDL levels and reduced HDL was significantly present (*p* < 0.05) in T2D patients, the complete absence of the *Ala12* allele in the population limited the assessment of the influence of this polymorphic variant on obesity and lipid abnormalities among T2D subjects.

## 5. Conclusions

The *Ala* mutant allele of *PPAR-γ2* was absent in this study and thus the Pro12Ala *PPAR-γ2* polymorphism may not be associated with obesity and/or T2D in this population. However, obesity and dyslipidaemia were associated with T2D. Our finding confirms previous findings around the sub-Sahara region and other African ethnic group which have shown *Ala12* allele to be totally absent or have a very low frequency in African populations.

## Figures and Tables

**Table 1:** Baseline and clinical characteristics of participants.

Characteristics	T2D Patients	ND Patients	*p*-Value
Age (years)	56.83 ± 1.21	49.03 ± 1.89	0.001
Height (m)	1.58 ± 0.01	1.61 ± 0.01	0.179
Weight (kg)	78.85 ± 3.42	71.52 ± 1.95	0.043
SBP (mmHg)	132.69 ± 2.65	132.86 ± 3.23	0.967
DSP (mmHg)	78.80 ± 1.36	81.76 ± 2.24	0.240
FBS (mg/dL)	166.71 ± 11.39	65.75 ± 3.79	0.000

Values are presented as Mean ± S.E.M. Age, weight and FBS are significantly higher (*p* < 0.05) in T2D patients. SBP = Systolic blood pressure; DSP = Diastolic blood pressure; FBS = Fasting blood sugar.

**Table 2:** Comparison of obesity and lipid abnormalities in patients.

Parameter	T2D Patients	*n* (%)	ND Patients	*n* (%)	*p*-Value
BMI (Kg/m^2^)	31.38 ± 1.41	31 (21.1)	27.81 ± 0.76	22 (15.0)	0.026
WC (cm)	100.15 ± 1.70	53 (36.1)	89.37 ± 2.25	32 (21.8)	<0.001
TC (mg/dL)	291.78 ± 28.90	58 (39.4)	159.43 ± 7.77	11 (7.5)	<0.001
TG (mg/dL)	241.33 ± 15.443	60 (40.8)	148.82 ± 7.59	31 (21.1)	<0.001
LDL (mg/dL)	212.40 ± 29.42	41 (27.8)	78.96 ± 7.96	9 (6.2)	<0.001
HDL (mg/dL)	33.55 ± 2.14	45 (30.6%)	62.74 ± 5.16	25 (17.0%)	<0.001

Values are presented as Mean ± S.E.M. *n* = the number of patients and prevalence (%) of obesity based on BMI and WC or number of patients with lipid abnormalities based on TC, TG, LDL, and HDL. BMI = Body mass index; WC = Waist circumference; TC = total cholesterol; TG = triglyceride; LDL = Low density lipoprotein; HDL = High density lipoprotein.

## References

[1] World Health Organization (2016). Global Report on Diabetes.

[2] Mokdad A.H., Bowman B.A., Ford E.S., Vinicor F., Marks J.S., Koplan J.P. (2001). The continuing epidemics of obesity and diabetes in the United States. J. Am. Med. Assoc..

[3] World Health Organization (2018). Obesity and Overweight. http://www.who.int/mediacentre/factsheets/fs311/en/.

[4] Mooradian A.D. (2009). Dyslipidemia in type 2 diabetes mellitus. Nat. Clin. Prac. Endocrinol. Met..

[5] Goldberg I.J. (2001). Diabetic dyslipidemia: Causes and consequences. J. Clin. Endocrinol. Met..

[6] Boden G., Shulman G.I. (2002). Free fatty acids in obesity and type 2 diabetes: Defining their role in the development of insulin resistance and beta-cell dysfunction. Eur. J. Clin. Investig..

[7] Blaschke F., Takata Y., Caglayan E., Law R.E., Hsueh W.A. (2006). Obesity, peroxisome proliferator activated receptor, and atherosclerosis in type 2 diabetes. Arterioscler. Thromb. Vasc. Biol..

[8] Desvergne B., Wahli W. (1999). Peroxisome proliferator-activated receptors: Nuclear control of metabolism. Endocr. Rev..

[9] Chawla A., Schwarz E.J., Dimaculangan D.D., Lazar M.A. (1994). Peroxisome proliferator-activated receptor (PPAR) gamma: Adipose-predominant expression and induction early in adipocyte differentiation. Endocrinology.

[10] Tavares V., Hirata R.D., Rodrigues A.C., Monte O., Salles J.E., Scalissi N., Speranza R.C., Hirata M.H. (2005). Association between Pro12Ala polymorphism of the *PPAR-G2* gene and insulin sensitivity in Brazilian patients with type-2 diabetes mellitus. Diabetes Obes. Metab..

[11] Khan M.A., St Peter J.V., Xue J.L. (2002). A prospective, randomized comparison of the metabolic effects of pioglitazone or rosiglitazone in patients with type 2 diabetes who were previously treated with troglitazone. Diabetes Care.

[12] Stumvoll M., Haring H. (2002). The peroxisome proliferator–activated receptor-γ2 Pro12Ala polymorphism. Diabetes.

[13] Yen C.J., Beamer B.A., Negri C., Silver K., Brown K.A., Yarnall D.P., Burns D.K., Roth J., Shuldiner A.R. (1997). Molecular scanning of the human peroxisome proliferator activated receptor gamma (hPPAR gamma) gene in diabetic Caucasians: Identification of a Pro12Ala PPAR gamma 2 missense mutation. Biochem. Biophys. Res. Commun..

[14] Vigouroux C., Fajas L., Khallouf E., Meier M., Gyapay G., Lascols O., Auwerx J., Weissenbach J., Capeau J., Magre J. (1998). Human peroxisome proliferator activated receptor-gamma2: Genetic mapping, identification of a variant in the coding sequence, and exclusion as the gene responsible for lipoatrophic diabetes. Diabetes.

[15] Deeb S.S., Fajas L., Nemoto M., Pihlajamaki J., Mykkanen L., Kuusisto J., Laakso M., Fujimoto W., Auwerx J. (1998). A Pro12Ala substitution in PPAR*γ*2 associated with decreased receptor activity, lower body mass index and improved insulin sensitivity. Nat. Genet..

[16] Mori H., Ikegami H., Kawaguchi Y., Seino S., Yokoi N., Takeda J., Inoue I., Seino Y., Yasuda K., Hanafusa T. (2001). The Pro12 3Ala substitution in PPAR-γ is associated with resistance to development of diabetes in the general population: Possible involvement in impairment of insulin secretion in individuals with type 2 diabetes. Diabetes.

[17] Scacchi R., Pinto A., Rickards O., Pacella A., De Stefano G.F., Cannella C., Corbo R.M. (2007). An analysis of peroxisome proliferatoractivated receptor gamma (PPAR-gamma2) Pro12Ala polymorphism distribution and prevalence of type 2 diabetes mellitus (T2DM) in world populations in relation to dietary habits. Nutr. Metab. Cardiovasc. Dis..

[18] Ben Ali S., Ben Yahia F., Sediri Y., Kallel A., Ftouhi B., Feki M., Elasmi M., Haj-Taieb S., Souheil O., Sanhagi H. (2009). Gender-specific effect of Pro12Ala polymorphism in peroxisome proliferator-activated receptor gamma-2 gene on obesity risk and leptin levels in a Tunisian population. Clin. Biochem..

[19] Mato E., Pokam-Fosso P., Atogho-Tiedeu B., Noubiap J., Evehe M., Djokam-Dadjeu R., Donfack O., Ngwa E., Guewo-Fokeng M., Mbacham W. (2016). The Pro12Ala polymorphism in the *PPAR-γ2* gene is not associated to obesity and type 2 diabetes mellitus in a Cameroonian population. BMC Obes..

[20] WHO-IDF (2014). Definition and Diagnosis of Diabetes Mellitus and Intermediate Hyperglycemia: Report of a WHO/IDF Consultation. http://www.idf.org/webdata/docs/WHO_IDF_definition_diagnosis_of_diabetes.pdf.

[21] Trinder P. (1969). Determination of blood glucose using 4-aminophenazone as oxygen acceptor. J. Clin. Pathol..

[22] Allain C.C., Poon L.S., Chan C.S., Richmond W. (1974). Total cholesterol assay. Clin. Chem..

[23] Esders T.N., Michira C.A. (1997). Triglyceride estimation. J. Biol. Chem..

[24] Grove T.H. (1979). Grove’s method of high density lipiptotein estimation. Clin. Chem..

[25] Friedwald W.T., Levy R.I., Fredrickson D.S. (1972). Estimation of the concentration of low-density lipoprotein cholesterol in plasma without use of preparative ultracenrifugation. Clin. Chem..

[26] International Diabetes Federation (IDF) (2013). IDF Diabetes Atlas.

[27] Arnaiz-Villena A., Fernandez-Honrado M., Areces C., Enriquez-de-Salamanca M., Abd-El-Fatah-Khalil S., Coca C., Arribas I., Algora M., Rey D. (2013). Amerindians show no association of *PPAR-γ2* gene *Ala12* allele and obesity: An “unthrifty” variant population genetics. Mol. Biol. Rep..

[28] Ruiz-Narvaez E. (2005). Is the *Ala12* variant of the *PPAR**γ* gene an “unthrifty allele”?. J. Med. Genet..

[29] Stumvoll M., Wahl H.G., Loblein K., Becker R., Machicao F., Jacob S., Haring H. (2001). The Pro12Ala polymorphism in the peroxisome proliferator–activated receptor-2 gene (*PPAR**γ2*) is associated with increased antilipolytic insulin sensitivity. Diabetes.

[30] Stumvoll M., Haring H. (2001). Reduced lipolysis as possible cause for greater weight gain in subjects with the Pro12Ala polymorphism in *PPAR**γ2*. Diabetologia.

[31] Danquah I., Othmer T., Frank K.L., Bedu-Addo G., Schulze M.B., Mockenhaupt F.P. (2013). The *TCF7L2 rs7903146* (T) allele is associated with type 2 diabetes in urban Ghana: A hospital-based case–control study. BMC Med. Genet..

[32] Yao Y.S., Li J., Jin Y.L., Chen Y., He L.P. (2014). Association between *PPAR**γ2* Pro12Ala polymorphism and obesity: A meta-analysis. Mol. Biol. Rep..

